# Severe Left Ventricular Failure Unmanageable by Impella 5.0

**DOI:** 10.7759/cureus.47882

**Published:** 2023-10-28

**Authors:** Akiko Mano, Tomohiro Murata, Akitoshi Inui, Mitsuhiro Kawata, Shunei Kyo

**Affiliations:** 1 Cardiothoracic Surgery, Tokyo Metropolitan Geriatric Hospital and Institute of Gerontology, Tokyo, JPN

**Keywords:** extracorporeal membrane oxygenation support, impella device, pulmonary hypertension, device therapy in heart failure, st-elevation myocardial infarction (stemi)

## Abstract

Mechanical circulatory support can be beneficial for patients with cardiogenic shock. Of these, the Impella has recently become the first-line device due to its feasibility, minimal invasiveness, and efficacy. We had a 58-year-old male with acute myocardial infarction followed by cardiogenic shock. We initially placed the patient on intra-aortic balloon pumping, which was switched to Impella 2.5 and could stabilize him. Unfortunately, the Impella 2.5 device suddenly stopped on the fifth day, thus, we tried to manage him by inotropes. However, his condition gradually deteriorated, so we applied Impella 5.0. Although his systemic circulation could be maintained, severe pulmonary hypertension persisted on Impella 5.0. He developed flash pulmonary edema, thus, we emergently added venoarterial extracorporeal membrane oxygenation on Impella 5.0 (ECPELLA). Then, we removed Impella 5.0 and changed peripheral venoarterial extracorporeal membrane oxygenation to central venoarterial extracorporeal membrane oxygenation. In this central venoarterial extracorporeal membrane oxygenation, we inserted the cannulas in the pulmonary artery and the left ventricle in addition to the usual cannulas in the ascending aorta and the right atrium. We aimed to control pulmonary arterial blood flow for lung protection as well as left ventricular unloading by this modification. However, his cardiac function showed no signs of recovery, and his lung condition showed further exacerbation. He was complicated by fungal sepsis and finally died of multi-organ failure. Although the Impella is an option, it is crucial to evaluate patients’ condition carefully and to escalate the device, if needed, without delay.

## Introduction

Mechanical circulatory support (MCS) is a fundamental tool for cardiogenic shock (CS). MCS is applied as a bridge to a decision, as a bridge to the next therapy, or until native heart recovery [1]. The Impella is a micro axial pump that entrains blood from the left ventricle (LV) and pumps it into the ascending aorta (AA). The Impella 5.0/5.5 can provide full LV support [2]. The Impella can maintain adequate systemic perfusion, reduce myocardial oxygen demand and consumption, and decrease LV volumes and pressures, which accelerate LV recovery [1,3]. Moreover, it can be inserted percutaneously [1-3]. Because of these characteristics, the Impella is increasingly used. Unfortunately, most advanced cases of LV failure (LVF) cannot be supported by the Impella. We encountered a patient with progressive LVF that was unable to be managed by the Impella 5.0. Although we switched the Impella to hybrid venoarterial extracorporeal membrane oxygenation (VA-ECMO), we could not rescue the patient. Accurate and timely MCS application is important for CS.

## Case presentation

**Figure 1:**
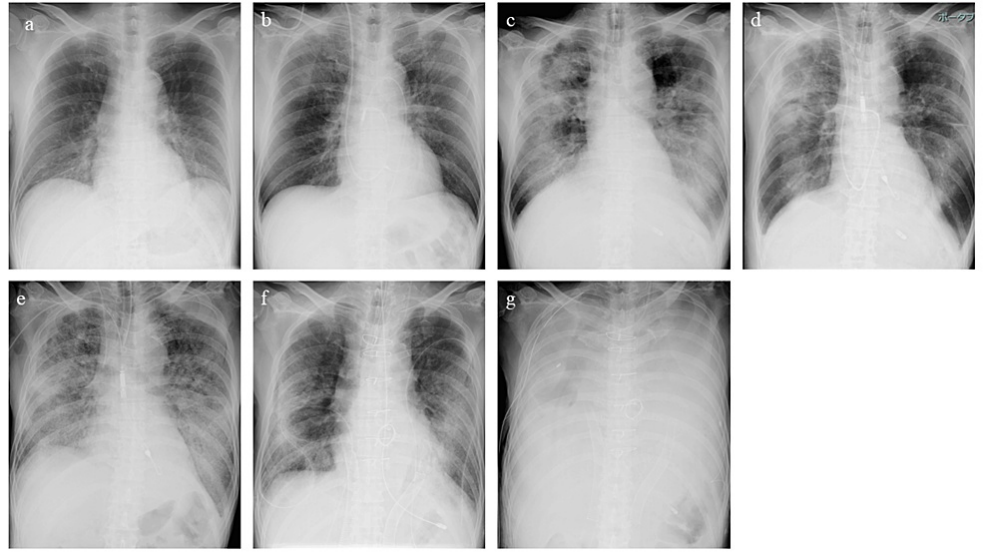
Changes in chest X-ray findings a; on admission, b; after Impella 2.5, c; before Impella 5.0, d; after Impella 5.0, e; before ECPELLA (adding veno-arterial extracorporeal membrane oxygenation (VA-ECMO) on Impella 5.0), f; after hybrid VA-ECMO, g; after extubation hybrid VA-ECMO

A 58-year-old man with a past medical history of hypertension presented with chest pain that persisted for five hours. On admission, an electrocardiogram revealed an ST-segment elevation in leads V2-4. A chest X-ray showed mild congestion (Figure 1a).

An echocardiogram revealed akinesis of the apex, anterior wall, lateral wall, and septum with a reduced ejection fraction (EF) of 30%. He was diagnosed with ST-segment elevation myocardial infarction (STEMI) and underwent emergent cardiac angiography (CAG). The CAG showed a total occlusion of the proximal left anterior descending artery. A subsequent percutaneous coronary intervention (PCI) was performed and thrombolysis in myocardial infarction (TIMI) grade 3 flow was achieved. His maximum level of creatinine kinase (CK) was 6,460 IU/L six hours after PCI. Shortly after PCI, he developed CS, so we started dobutamine and placed him on intra-aortic balloon pumping (IABP) that was inserted from the right femoral artery. However, his condition was unstable on IABP; thus, we switched IABP to Impella 2.5, which was replaced by a guide wire from the same right femoral artery, on the third day. His hemodynamics were stabilized on Impella 2.5 at the P-5 support level. He could be weaned off dobutamine the next day. His lung congestion was also improving (Figure 1b).

**Figure 2:**
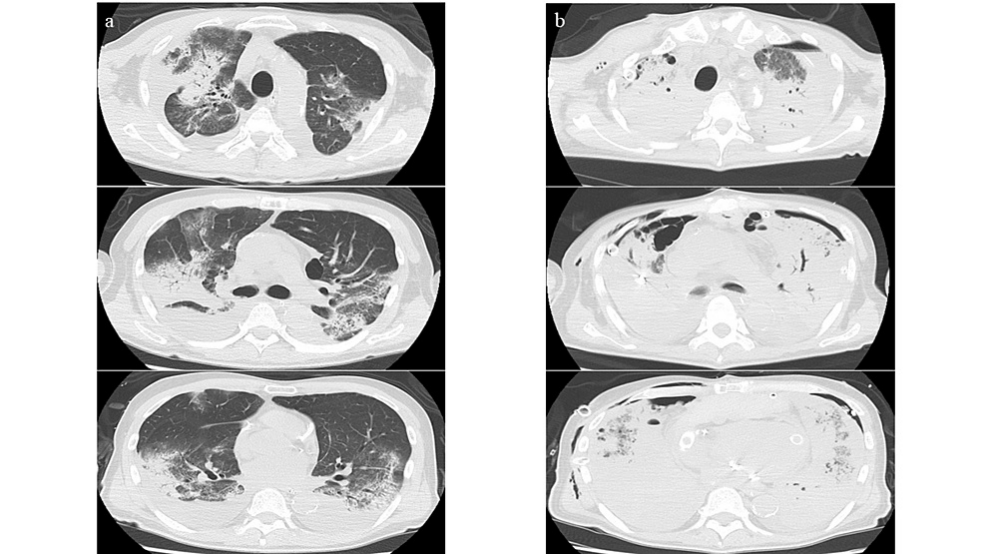
Changes in chest CT findings a; before Impella 5.0, b; after extubation on hybrid VA-ECMO CT; computed tomography, VA-ECMO; veno-arterial extracorporeal membrane oxygenation

At this time, his cardiac index (CI) was 2.5 L/min/m^2^ and pulmonary capillary wedge pressure (PCWP) was 16 mmHg. However, the Impella 2.5 suddenly stopped on the fifth day. We immediately started dobutamine. His hemodynamics were maintained without signs of end-organ function deterioration on a moderate dose of dobutamine. However, his lung congestion worsened each day, even with aggressive diuresis. Unfortunately, he developed pneumonia. We increased dobutamine and added milrinone, but lung congestion worsened, and his oxygen demand increased. On the twelfth day after Impella 2.5 removal, we intubated him. We increased inotropes and continued aggressive diuresis; however, LV function still severely deteriorated without restoration, and lung congestion persisted (Figure 1c, Figure 2a).

At this point, on dobutamine of 4 μg/kg/min and milrinone of 0.25 μg/kg/min, his blood pressure was 110/62 mmHg, pulmonary artery pressure was 34/21 mmHg, pulmonary capillary wedge pressure (PCWP) was 15 mmHg, central venous pressure was 7 mmHg, and CI was 2.7 L/min/m^2^. Although his hemodynamic parameters were acceptable, he needed a decent amount of inotropes and showed persistent lung congestion. We thought that more aggressive LV unloading would be beneficial to improve lung congestion and accelerate LV recovery. Therefore, we inserted the Impella 5.0 via the right subclavian artery on the seventh day after Impella 2.5 removal. Initially, we set the Impella 5.0 to a P-9 support level so that we could stop inotropes the next day. His hemodynamics were maintained, with stable end-organ function and improved lung congestion (Figure 1d).

However, hemolysis was detected on the third day. We checked the echocardiogram, which showed no obvious Impella disposition, reduced LVEF of 30%, and moderate mitral regurgitation (MR). We changed the support level to P-7, and then, hemolysis was resolved. At this support level, his hemodynamics were stable with a CI of around 2.5 L/min/m2, but his pulmonary hypertension (PH) remained; his PCWP was middle 20 mmHg. His oxygenation and lung congestion gradually improved, so we performed extubation on the fourth day. After extubation, his hemodynamics showed no change, but his respiratory condition worsened. Two days after extubation, he developed flash pulmonary edema with massive foamy bloody sputum immediately after defecation (Figure 1e).

We performed emergent intubation; but could not stabilize his respiratory condition. Therefore, we started VA-ECMO (ECPELLA). The cannulas were inserted from the left femoral artery and vein. His hemodynamics and oxygenation were stabilized on ECPELLA, but cardiopulmonary function severely deteriorated with no sign of restoration; thus, we switched ECPALLA to hybrid VA-ECMO on the ninth day (Figure 1f).

As we previously reported, we could maintain sufficient systemic circulation, perform biventricular unloading adequately, and control pulmonary artery PA blood flow (PABF) by this unique system; the cannulas to the pulmonary artery and to the left ventricle in addition to the cannulas to the ascending aorta and to the right atrium [4]. We selected this system because his RV function was also reduced due to persistent PH, and we needed to control his PABF to ameliorate pulmonary hemorrhage. On hybrid VA-ECMO, he was stabilized, but again, he showed no signs of recovery. On the sixth day on hybrid VA-ECMO, he developed pneumothorax, so we were forced to perform extubation. Unfortunately, his lung condition quickly deteriorated after extubation. Most alveoli were filled with fluid and had almost no air spaces, although we controlled the renal arterial blood flow (RABF) (Figure 1g, Figure 2b).

Shortly after that, he developed a fungal infection, followed by multiorgan failure, and died on the seventeenth day of hybrid VA-ECMO (after the forty-ninth day after STEMI).

## Discussion

CS is a life-threatening condition that is induced by a wide range of heart diseases. MCS is an essential means of CS management. The Impella has recently been accepted as the first-line device for CS because of its feasibility and minimal invasiveness. It is a micro-axial pump that is implanted percutaneously, across the aortic valve and directed backward [1]. Smaller devices like the Impella 2.5 and Impella CP can be inserted percutaneously via the femoral artery. Larger devices like the Impella 5.0 and Impella 5.5 require a surgical cut-down but are much less invasive than a left ventricular assist device (LVAD) [1-3]. It unloads the LV, reduces LV work, and accelerates native LV recovery while providing adequate systemic circulation [1,3]. The Impella 5.0 and 5.5 are perceived to provide full LV support [2], so they have recently replaced temporary LVAD. Ramzy D et al. reported an early experience of Impella 5.5 for CS; of their 55 patients, 35 patients were successfully weaned off the device with recovery, 11 patients were bridged to another therapy, and the overall survival was 83.6% [1]. Kennel et al. reported excellent outcomes of Impella 5.5 when used to manage CS [5]. They showed that more than 75% of the 14 patients in the study were successfully bridged to further advanced therapies or weaned from the device due to recovery. These early reports show that the Impella is effective in managing CS.

In our case, after inserting the Impella 5.0, the patient could be weaned from inotropes and his systemic circulation was adequately maintained. His oxygenation also improved; thus, he could be extubated. However, PH persisted even after aggressive diuresis. He also had more than moderate MR. Eventually, he developed flash pulmonary edema and needed MCS escalation. We thought that these findings indicated that the Impella 5.0 could provide considerable cardiac output; but could not unload the LV sufficiently. This may be related to incomplete Impella support power. We reduced the Impella support level due to hemolysis, so we were unable to increase the support level to a maximum level. He had an acute myocardial infarction (AMI), thus, his LV was not dilated; the LV diastolic diameter was 46 mm. This LV size might be disadvantageous to increase the Impella support level. We also suspected that MR negatively impacted LV unloading, and this instance of MR might pertain to Impella’s position although we could not confirm the exact position due to poor echo imaging. Kennel et al. reported that the Impella was entangled in the mitral subvalvular apparatus and required removal and replacement [5]. Lung damage due to pneumonia may be related to PH; however, his PCWP was also high; therefore, we believed that insufficient LV unloading had the greatest impact on his PH. His LV function might have been too deteriorated to be sufficiently supported by Impella 5.0. We should have escalated MCS earlier, specifically, we should have re-inserted the Impella 2.5 just after the initial Impella 2.5 stopped or should have applied the Impella 5.0 sooner, or should have considered switching the Impella 5.0 to extracorporeal LVAD earlier. However, the patient was not a candidate for heart transplantation due to social issues, thus, we hesitated in MCS escalation, which led to an unfavorable delay. It might be better to add aggressive medication for PH combined with Impella 5.0.

Schurtz et al. compared the outcomes of homogenous patients with CS who received an Impella or were on VA-ECMO [6]. They concluded that Impella was associated with a poor survival rate and a greater need for MCS escalation. Based on these data, they suggested that the Impella could be considered in less severe CS patients. This finding is consistent with our experience. Although the Impella is a useful tool, candidates should be selected carefully, and device escalation should be performed without delay, if needed.

## Conclusions

We experienced a case of severe LVF, which was difficult to manage with Impella 5.0. Although the Impella could be a potential option for CS, careful patient selection would be crucial to utilize this attractive device.
